# “A Free People, Controlled Only by God”: Circulating and Converting Criticism of Vaccination in Jerusalem

**DOI:** 10.1007/s11013-020-09705-2

**Published:** 2021-02-04

**Authors:** Ben Kasstan

**Affiliations:** 1 Centre for Health, Law & Society, University of Bristol, Bristol, UK; 2 Department of Sociology and Anthropology, Hebrew University of Jerusalem, Jerusalem, Israel

**Keywords:** Conversion, Jerusalem, Religion, USA, Vaccination

## Abstract

This paper explores how criticism surrounding the ethics and safety of biomedical technologies circulates and ‘converts’ through global–local religious encounters, producing new claims of moral opposition and rights to religious freedom. The paper is concerned with the question of what rhetorical devices make vaccine safety doubt relevant to religiously Orthodox settings and what implications arise? Based on an ethnographic study of vaccine decision-making and non-vaccination advocacy in Jerusalem, the paper examines how opposition is forged amidst evolving global–local encounters and relations. The data reveal how Christian activists attempt to engender ethical and moral opposition to vaccination among American Orthodox Jews in Jerusalem by ‘converting’ public criticism around safety into a religious discourse of bodily governance. Pinpointing how critiques of biomedical technologies discursively ‘convert’ offers a conceptual template in anthropology to chart how counter-positions are formed and transformed amidst evolving tensions between biomedical and religious cosmologies.

## Introduction

This paper explores how criticism surrounding the ethics and safety of biomedical technologies circulates and ‘converts’ through global–local religious encounters, producing new claims of moral opposition and rights to religious freedom. In what follows, I use the case of vaccination to demonstrate how popular refusal of biomedical technologies that are deemed “unsafe” become voiced in a rhetoric of bodily devotion to God. The paper is concerned with the question of what rhetorical devices make vaccine safety doubt relevant to religiously Orthodox settings and what implications arise? My approach focuses analytical attention on how scientific truth-claims around safety and disability are deployed to present non-vaccination as a religious right, thus signifying how secular and non-secular sites in biomedicine converge (Roberts 2016).

Anthropologists have been prolific in demonstrating how reproductive biomedicine offers a wealth of insights into how technologies are negotiated among religious movements through techniques of discursive transformation (Kahn 2006; Inhorn 2012; Roberts 2006). Vaccination, by contrast, is less frequently examined in these sets of debates, despite evidence to suggest that religious authorities sanction and oppose vaccines in diverse ways (Kasstan 2019; 2020; Renne 2010). Anthropologists and sociologists tend to focus on non-vaccination among educated, middle class and white parents in the global north (Reich 2014; Sobo 2015). Yet, I suggest that religiously Orthodox minorities require an analytical shift; they are seemingly protected from the pressures of the ‘secular’ world, but incorporate its messages amidst evolving global–local encounters and via processes of discursive ‘conversion.’ Public criticism surrounding the ethics and safety of biomedical technologies circulates across physical and social borders, but also converts by undergoing a religious transformation of ideas and rationales.^1^

This paper draws on an ethnographic study into vaccine decision-making in Jerusalem. The majority of parents I met were Orthodox Jews from North America, which enabled me to trace how ideas of vaccination circulate in a ‘global religious network’ (Taragin-Zeller and Kasstan 2020), which has been linked to persistent outbreaks of measles (see McDonald et al. 2019). My findings show that American Christian activists culturally, religiously, historically and politically ‘convert’ and make non-vaccination messaging relevant to ‘local moral worlds’ (Kleinman 1992). I use the term ‘conversion’ to signify how American Christian activists frame vaccination as a general theological issue in their encounters with American Orthodox Jews, and as an attempt to engender vaccine opposition as a form of moral regulation. In the most extreme form, non-vaccination messaging draws on the Holocaust (*Shoah*) and the ‘politics of threatened life’ in Israel (cf. Ivry 2009) to present ‘vaccine questioning’ as necessary for Jewish preservation. Jewish Parents localize the conversion process by situating their vaccine decisions into more specific frameworks around the body in Judaism. My ethnographic data reveal how self-protective minorities are not ‘immune’ from the global circulation of vaccine messaging, which is forging new entanglements of vaccine opposition that are underpinned by shared claims of Divine governance over bodies.

Anthropologists have examined how modern technologies engender a ‘leap of faith’ and raise everyday dilemmas of religious transgression as well as transformation (Fader 2020; Stadler 2009; Taragin-Zeller in press). Listening to how common criticism of vaccine safety circulates and ‘converts’ captures how non-vaccination becomes framed as an act of devotion to God, where religious law is interpreted in conflicting ways to the broad acceptance of vaccines by most religious authorities. Pinpointing how critiques of biomedical technologies circulate and ‘convert’ offers anthropologists a conceptual template to identify how counter-positions are formed and transformed amidst evolving tensions between biomedicine and religion.

### Technologies that Travel

The globalization of biomedical technologies, especially regarding reproductive health, has raised dramatic implications for religious groups and movements. A conceptual departure, however, can be traced when biomedical technologies are envisaged in accordance with religious standpoints, or used despite social and religious sanctions.

As an example of the former, Risa Cromer (2019) demonstrates how evangelical Christians in the US appropriate the biomedical discourse of embryo ‘donation’ and offer ‘embryo adoption’ services as an attempt to ‘rescue’ leftover gametes from ‘frozen orphanages.’ Embryo ‘adoption’ then serves as a strategy to deliver technologies in line with ‘Pro-Life’ philosophies. In a similar vein of conceptual transformation, reproductive technologies that enable Orthodox and Haredi Jews in Jerusalem and New York to realize and achieve their reproductive duties or labor become ‘sacralized as a blessing from above and part of the divine plan’ (Teman, Ivry and Goren 2016:276; Kahn 2006).

Concerning the latter, Senegalese Muslims can desire prenatal genetic testing and selective abortion to avoid birthing a child with sickle cell disease, with the ‘sin’ of abortion rationalized on the basis that a ‘human thing is not [yet] a human being’ (Fullwiley 2004:176). While relying on the Quran as an ethical framework, Senegalese Muslims perceive abortion as a technique of preventing genetic disease in ways that reveal a moralization of technology (Fullwiley 2004). In India, where sex-selective abortion is criminalized, medical providers view the practice as a ‘social service’ amidst a ‘social fact’ of gender inequality and when the ability to bear a male heir can secure the position of Hindu and Muslim women in the household (Unnithan-Kumar 2010). It has therefore been argued that ‘technologies in themselves do not bring about social transformation but it is in how they are made socially meaningful that their power lies’ (Unnithan-Kumar 2010:163).

These diverse examples share a common interest in how globalized technologies travel and produce situated responses, yet I am interested in how *criticism* of the ethics and safety of technologies circulate between contexts. I instead take vaccination as an opportunity to push forward anthropological debates on how global circulation of biomedical technologies bring discursive transformations that depart from the positions of religious authorities.

Vaccines are a uniquely routinized biomedical technology insofar as they aim to reach every child on the planet, enabled by a particular ‘technocracy,’ or in other words, the ‘governance regimes devoted to ensuring timely delivery and uptake’ (Leach and Fairhead 2007:7). For this reason, state vaccination programs have been conceptualized as eminently ‘political projects that presume to shape the immunity of whole populations’ (Greenough, Blume and Holmberg 2017:1). While vaccines work to prevent diverse forms of disease, disability and death, parents refuse vaccination due to concerns of adverse reactions (Casiday 2007; Kaufman 2010; Poltorak et al. 2005; Reich 2014; Sobo 2015).

Scholars increasingly examine vaccination as a social tension that produces and re-produces rhetoric of responsible parenting. Considering the public ‘good’ of vaccination as a mechanism of social immunity or population protection, public (health) discourse is replete with derogatory views of ‘anti-vaxxers’—which ‘depends upon a particularly damaging kind of group character assassination’ (Hausman 2019:13). Pro-vaccine activism ‘reinforces the mainstream and counters digression from it’ (Vanderslott 2019:60). These tensions are all the more fraught at the time of writing, as ‘hesitancy’ surrounding vaccination has emerged as a major threat to global health, commensurate with the dangers posed by climate change and antimicrobial resistance (WHO 2019).

Vaccine ‘hesitancy’ is a relatively new public health term that attempts to acknowledge parental concerns, and depart from the disparaging, pervasive and collective term ‘anti-vaxxers.’ On the other hand, however, ‘hesitancy’ may not capture how parents feel confident about their decisions to selectively decline or outright refuse vaccinations—as this paper signifies. I maintain that social scientists should engage with parents who report anecdotal changes in their children, but also the rhetoric used by non-vaccination activists and the situated implications that arise in ‘local moral worlds.’

Few anthropological studies, however, grapple with the apparent issue of ‘religious opposition’ to vaccination, despite global measles outbreaks being attributed to religious minorities and movements—including Orthodox Jews, Dutch Protestants and Amish. Anthropologists tend to share a common analytical approach of examining how religious authorities influence the decisions of devotees, particularly in the global south (Feldman-Savelsberg, Ndonko and Schmidt-Ehry 2000; Renne 2010). I instead shift the analytical gaze to the circulation and “conversion” of rhetorical techniques to present vaccines as disabling and disrupting of Divine governance over bodies.

### Migration and Moral Regulation

The Jewish parents I met in Jerusalem offer an ideal case study to examine the circulation and conversion of vaccine safety criticism as they cross two key borders as migrants and as ‘returnees’ to Judaism. Firstly, they are regarded vernacularly as *Olim* (‘immigrants’) or *Anglo-Saxim* (from ‘Anglo-Saxon’ countries) and are of particular interest because they mark a transition from a ‘Diaspora’ or minority positionality to migrants in a Jewish majority setting. Anthropologists examining migration to Israel tend to focus on the role of navigating bureaucracy (Egorova 2015; Kravel-Tovi 2017) and experiences of public health exclusion (Seeman 1999), especially when migrants are not considered Jewish according to the matrilineal definition upheld by the State. The experience of *Olim* from North America is different in so far as many identify as Orthodox, hold cultural-capital as English speakers and often Modern Hebrew—and thus have an advantage to assimilate into the Israeli body politic (e.g., Waxman 1989). Moreover, as I go on to explain, this particular migration brings ideas of moral regulation, especially abortion, which reflects public discourse in the US and departs from the broader Jewish–Israeli population (Levine 1994).

Secondly, common to the parents I met is that they “returned” to Orthodox Judaism and made conscious decisions to live according to varying and heightened standards of Jewish law (*ba’alei tshuvah)*. They are situated in a ‘cultural borderland’ (Benor 2012) that enables an integration of navigation skills—especially regarding the non-Orthodox world and their practice of Judaism. This means that *ba’alei tshuvah* are particularly well placed to situate common vaccination concerns in the ‘local moral world’ of religious Orthodoxy, and their positionality sheds a unique light on circulation and conversion of non-vaccination advocacy.

While diverse, the Orthodox and Haredi (‘ultra-Orthodox’) Jewish parents I met share a commitment to living in accordance with the teachings derived from the Hebrew Bible (*Tanakh*) as well as a compendium of rabbinic literature, commentary and rulings—albeit to varying interpretations, leniencies and stringencies. Whereas Orthodox Jews attempt to reconcile piety and professional employment, Haredi Jews can be characterized by a general pursuit of ‘immunity’ or self-protection from external influences—as an attempt to maintain purity from danger (cf. Douglas 2002). The term Haredi means to be God-fearing, or to tremble in awe of God, capturing how decisions around bodily governance are made in close consultation with religious scripts (Stadler 2009).

The body of Jewish law (*halachah*) does not have a definitive position on vaccination, though rabbinic authorities widely interpret vaccines as permissible—if not mandated (Orthodox Union 2018). Yet, looking beyond the rabbinic acceptance of vaccination raises broader issues pertaining to pursuits of bodily protection, the role and reliability of religious authorities in health decision-making, and emerging articulations of rights to religious freedom.

I found that Christian activists were considered a form of ‘authoritative knowledge’ (Jordan 1997) and invited to non-vaccination advocacy events, which warrants a brief note on the emphasis of moral regulation in Christian evangelicalism. Evangelical Christianity is diverse (Coleman and Hackett 2015), though there is a shared belief ‘about their own sense of spirituality and their commitment to using it to change the world around them’ (Luhrmann 2012:13). Evangelical Christians are characterized, among many pursuits, by a morally regulatory regime, as well as a struggle to inscribe that regime in a given state’s legal order (de Almeida 2020). Such moral regulation of bodily practices has long been seen in relation to abortion in the US (Ginsburg 1988), as well as more recently following advancements in reproductive biomedicine (Cromer 2019). A number of Christian faith leaders in the US disagree with vaccinations that are cultured on human-cell lines, derived from aborted fetal tissues (Williams and O’Leary 2019). Historically, Christian missions used medicine as techniques to convert souls and save bodies, including Jews (Kasstan 2019), yet conversion takes on new meanings when attempting to act upon ideas of health and bodily governance.

### Measles, Vaccines and Orthodox Jews

From New York and Jerusalem to London and Brussels, Orthodox and Haredi Jews have raised public health concerns of ‘religious opposition’ to vaccination. Lower-level vaccination coverage among Jewish neighborhoods in London, New York and Jerusalem have led to persistent outbreaks of measles (Letley et al. 2018), and were directly implicated in the 2018–2019 measles epidemic in the United States. Cases of measles began to emerge among New York’s ‘Orthodox Jewish communities’ in the autumn of 2018, which were linked to unvaccinated people traveling between Israel and the United States (McDonald et al. 2019). The outbreaks that originated in New York’s Jewish neighborhoods were declared a public health emergency by April 2019 (Silverberg et al. 2019), and led to the US experiencing its highest cases of measles in 25 years—as was the case in Israel.

Influences on vaccine decision-making among Orthodox and Haredi Jews are diverse and conflicting, and extend far beyond the realm of religious law. Studies conducted with Orthodox and Haredi Jews in London note that ‘in a community relatively insulated from direct media influence, word of mouth is nevertheless a potent source of rumors about vaccination dangers, whose origin may lie in media scares’ (Henderson, Millett and Thorogood 2008:250). Yet, subsequent research has not uncovered any ‘cultural or religious anti-vaccination sentiment’ within Haredi Jews in London (Letley et al. 2018:4687). Amidst the 2018–2019 measles outbreaks in New York, however, the US Department for Health and Human Services (2019) implied that Haredi Jewish neighborhoods were being deliberately targeted by non-vaccination activists. Their position was that ‘A significant factor contributing to the outbreaks this year has been misinformation […] Some organizations are deliberately targeting these communities with inaccurate and misleading information about vaccines.’ In what follows I explore how non-vaccination advocacy becomes assimilated in Orthodox and Haredi Jews through processes of discursive transformation or religious ‘conversion.’

## Methods

To explore how opposition to vaccination circulates and ‘converts,’ I conducted a twelve-month study exploring vaccine decision-making in Jerusalem from October 2019 to September 2020. This paper draws on data from twenty-two semi-structured interviews and ethnographic research of two-linked events in November 2019, which hosted international non-vaccination activists from the USA and Europe.

The first event was organized by an advocacy group led by American Orthodox Jews in Israel, and was held in Jerusalem for English-speaking Orthodox and Haredi Jews. The second event was held in Tel Aviv and publically presented as the ‘first international conference for informed consent.’ This event was primarily for the Hebrew-speaking Israeli population, with English subtitles accompanying presentations in Hebrew, as well as Hebrew subtitles accompanying the messages of international presentors.

Twenty-two in-depth and in-person semi-structured interviews were conducted with parents (mainly mothers and two fathers) on vaccine decision-making. All participants lived in Jerusalem and the surrounding area, with one participant living in Israeli settlements in the Occupied Palestinian Territories. Interviews were typically conducted in family homes while parents cared for children, but also in cafes and synagogues when parents had to juggle work commitments and responsibilities. The paper specifically focuses on parents who selectively or completely refused vaccinations and either supported or attended the events.

The majority of participants had migrated from North America (seventeen), with the remaining participants originating from the UK, Canada and South Africa. They arrived in Israel either as individuals or with their growing families under Israel’s ‘Law of Return,’ which confers Israeli citizenship to anybody with at least one Jewish grandparent. All participants of this study were of an Ashkenazi (East European) background, which can be explained by the fact that Ashkenazi Jews form the dominant part of the Jewish populations in the USA, UK and South Africa. The parents I met described themselves in diverse terms, ranging from modern Orthodox, to Orthodox, *Dati Leumi* (Religious Zionist),^2^ and Haredi, which is a highly diverse sector and consists of multiple groups, each with their own religious leaders (rabbis), teachings, observances and ethnic origins. Whereas scholars of religion treat these groups as separate analytical categories in the case of Israel, I decided to work across these groups to capture the continuities and discontinuities in family-making decisions (see also Taragin-Zeller 2019).

I was able to approach participants whom I had previously encountered in a study of vaccine decision-making in the UK (Kasstan 2017, 2019), as well as snowball sampling techniques. Interviews were recorded using a digital audio recording device, when permission was granted, and detailed notes recorded. Recordings from interviews and participant observations in the field were transcribed verbatim, and analyzed based on emerging themes. To protect the identities of interview participants, I have replaced their names with pseudonyms. Ethical approval to conduct this study was obtained from the Ethics Committee of the Faculty of Social Sciences Review Board of the Hebrew University of Jerusalem.

### Vaccine-Injured Bodies

One November evening in 2019, I walked to a luxury hotel in Jerusalem for an event held ‘in support of the vaccine-injured community.’ A red carpet led me to a large room partitioning women from men (Hebrew, *mechitsah*), as is characteristic of Orthodox and ultra-Orthodox interpretations of Jewish law. I purchased my entry ticket for 30 (approximately $9) at the counter, and was handed a pamphlet entitled ‘The Vaccine Safety Handbook: An Informed Parent’s Guide’—which was produced in English and imported from the USA. As I flicked through the pamplet, I noticed that it was produced by PEACH (Parents Educating & Advocating for Children’s Health), an advocacy group that describes itself as promoting ‘vaccine choice,’ and as being organized by and for Orthodox and ultra-Orthodox Jews in the USA. Delicate ceramic Judaica were being sold at the counter, and a notice explained that they were the ‘handiwork’ of a man in his twenties who has lived with autism and epilepsy since being ‘injured by vaccines’ during infancy. Stalls were lined with non-vaccination advocacy information.

A (male) speaker, Gavriel asked the guests to take their seats, and the men and women moved to their respective sides of the room. The speaker went on to articulate how the perceived reality of vaccine damage had brought the evening’s guests together in pursuit of Divine solace. ‘We can give each other strength,’ he paused. ‘And in that *zchut* [merit] of tonight, of being there for another, *Hashem* [God] shall open all the gates, and give us all hope, v’*tikvah tovah v’derecheh* [the path of hope and goodness].’ The audience applauded emphatically.

A live band and four (male) youths took to the stage, and were introduced as ‘vaccine-injured children.’ The boys sang, in Hebrew, ‘*Heal us, Hashem* [God]*, and have mercy upon us,’* to which the audience applauded loudly. The event went on to host a video testimony of a mother and her vaccine-injured child, presentations by a physician on the symptoms of autism, on treating vaccine-injured children by a homeopath, an address by Jim Meehan (a practicing physician and Christian), finally, a lecture by the US non-vaccination activist Del Bigtree. Hundreds of people had joined by the time Bigtree made his address, making him the clear attraction for the American Orthodox Jewish attendees.

It is important to mention here that Bigtree drew widespread condemnation in 2019 when donning a Nazi-era Star of David amidst the US measles outbreaks (Sun 2019), modeling what Jews in Germany and Nazi occupied territories were forced to wear as part of a systematic process of persecution that culminated in the *Shoah.* While Bigtree’s method of presenting scientific truth-claims have been critiqued elsewhere (Bricker and Justice 2018), his appearance in Jerusalem demonstrates the targeting of non-vaccination advocacy to Jews as part of a global–local circulation activism.

I want to draw attention to how the evening featured tacit and targeted references to the *Shoah*, the Nazi genocide of at least six million Jews that holds a deeply rooted legacy in Jewish and Israeli collective identity. As the *Shoah* is central to Haredi society (Caplan 2002), the intentional deployment of rhetoric to a specific religious audience is underscored. Del Bigtree presented the Nuremberg Code on a Powerpoint slide alongside an iconic image of children in striped pyjamas behind the barbed wire of concentration camps—characteristic of the *Shoah*. The intention of displaying the Nuremberg Code was to present an accusation of deliberate failure on the part of public health services to provide full informed consent and freedom of choice when vaccines, Bigtree maintained, lack thorough safety assessment and governance.^3^ This ultimately led Bigtree to say that ‘you are part of a medical experiment that you never signed up for,’ which, considered alongside the image described above, is an explicit rhetorical link to Nazi experiments conducted on Jewish adults and children in Auschwitz-Birkenau. In so doing, Bigtree sought to make his bioethical claims historically relevant to Jewish audiences.

### Converging Inflections of Moral Opposition

Religion was frankly referenced as a reason to challenge what has been termed the global vaccination ‘technocracy’ (Leach and Fairhead 2007), or what Bigtree termed ‘the cult of vaccinology.’ Bigtree declared his upbringing and credentials to the audience as the son of a Minister, which situated his truth-claims in a biomedical-religious contest of bodily governance. ‘I find it fascinating,’ he asserted, ‘that we can question God, but we cannot question pharma’—to which the audience applauded. Vaccines were framed as adulterating bodies, which, according to Abrahamic cosmologies, remain the property of God:

We know what we’re doing. We’re allowing our children to be designed the way they were meant to be designed. In my mind, they’re created in the image and likeness of God [audience applause]. They’re created perfect [audience applause]. And I don’t understand how any of us are letting ourselves believe that there’s some sort of original sin upon our children the moment they’re born. *An original sin of disease that God messed up so badly, that we’ve got to get these kids 72 vaccines, to live on God’s earth*. Does that sound crazy to anybody else? [Audience applause and shout of ‘yes’]. *We cannot let history repeat itself again*, we are not property of our governments [audience applause], we are free people [audience applause], controlled only by God, himself. [My emphasis]

What is important is how vaccines are discursively presented by Christian activists as a biomedical claim of Divine failure; the decision to accept vaccinations implicitly means to doubt Divine intentions—which is otherwise highly transgressive for Haredi or ‘God-fearing’ Jews. By deploying the (explicitly Christian) doctrine of ‘original sin,’ a tactic emerges of transforming ‘secular’ critique of vaccine safety in a generalized language of religious morality and Divine devotion. Not specific to Bigtree, Jim Meehan explicitly asserted that ‘we must rise to protect out religious freedoms.’ Using the example of vaccines cultured on human-cell lines, Meehan went on to criticize informed consent around vaccination: ‘as a Christian, I deem this abominable and horrific. Were we not deprived of full informed consent at the time my children were vaccination, we never would have consented to their defilement by some secret, sinister, satanic ritual […].’

More broadly, non-vaccination activists in the US advocate for ‘vaccine liberty’ by discursively pitting compulsory vaccination mandates as ‘government overeach’ and situating their activities in a historical tradition of pursuing civil rights (Reich 2018a). Yet, when looking at how non-vaccination activism intersects with religion, a picture emerges where the pursuit of civil rights morphs into a call to protect rights to religious freedom.

In another breath Bigtree reminds the Jewish audience of the *Shoah* as a tangible threat, insinuating that Jews are only safe under Divine authority. Similarly, Don Seeman observed how Israeli-Ethiopians deployed references to the *Shoah* as a rhetorical device amidst public health conflicts over blood donation, casting the former ‘in the role of the dangerous enemies of the Jewish people’ (1999:165). Drawing on such tropes in the case of vaccines instead signifies how contesting state intervention and governance over the social body is presented as necessary to avoid a repetition of a devastating collective trauma. The Jewish participants of the room, he asserted, should, never again, have to see their history repeated.

The attempt to cultivate a vaccine damaged camaraderie becomes a salient reflection of the ‘politics of threatened life’ (Ivry 2009), with a social-history of threat intentionally deployed to inspire non-vaccination. In this regard, the global–local circulation of non-vaccination messaging reflects how American influenced “pro-life” organizations operate in Israel and deploy American Christian tones and terminology (Steinfeld 2015; Levine 1998). The leading anti-abortion voice in Israel, ‘Efrat,’ has long concerned itself with declining Jewish birth rates and demographic anxieties vis-à-vis Palestinian citizens of Israel since its inception in the 1960s (see Raucher 2020). Yet, commentators have observed a clear discursive shift towards fetal rights to life in the organization’s rhetoric. As Noga Morag Levine (1994:319–320) notes, ‘The American anti-abortion movement was quite clearly the inspiration for this change, whose conduit appears to have been American Orthodox immigrants who became involved in Israeli abortion politics.’ Thus non-vaccination is continuous with broader forms of moral regulation that circulate, convert and converge through migration.

### Assimilating Messaging

Meeting parents who did attend the events enabled me to understand what information they were seeking, and the implications of Bigtree’s address. Raizel (age 48) had moved to Jerusalem from the US over 20 years ago while taking the decision to ‘return’ to Judaism as a (*ba’alat teshuvah*),^4^ and raise her five children according to the heightened standards of Jewish law that religious Orthodoxy entails. Reflecting on the Jerusalem event, Raizel recalled how ‘300 people came, which was just so validating to see, some people I knew from other times in life and just regular people who became religious like me.’ The evening’s draw, then, was the articulation of an identity around vaccine-damage that was voiced emically as a collective or community. In Raizel’s words, ‘some of them have had vaccine-injured kids, but we’re all like no way. No way should we be vaccinating our kids.’

When I asked how she perceived references to the *Shoah*, in Bigtree’s activism, Raizel said she ‘didn’t agree,’ but nonetheless reiterated that the important message to draw was the freedom to make informed and safe decisions. While I did not find evidence of the *Shoah* having a legacy on engagement with public health services among the collective of religiously Orthodox parents in this study, I did encounter a perception that Del Bigtree was seeking to prevent the ‘subjugation’ of Jews and that ‘authority’ needed to be questioned (as I go on to discuss). To quote Maya (age 76), who was herself a Polish child survivor of the *Shoah*:

Bigtree was showing people what could happen if we go to the direction of being subjugated by evil, that there are people in this world who want to control everyone. I hope Hashem will not let the world go that way. We can’t fall into being too obedient to authority.

Similarly, Tehilla (age 67) described her journey from New York to *Eretz Yisrael*, as she put it, using the Biblical term for the ‘land of Israel,’ and the decisions she made when raising her seven children.^5^ She attended the Tel Aviv conference to gather information and support advocates who she perceived to be persecuted:

I went to that all day conference [in Tel Aviv], and I met people there. I wanted to get the information, because to me, some of these people are heroes – these scientists that are speaking out, and losing their medical degree, their license, just because they’re speaking the truth.

In attending the advocacy events, Raizel and Tehilla were explicit in pursuing scientific information or ‘truths,’ which were otherwise perceived to be marginalized by the dominant and pro-vaccine contingent. Forms of ‘secular knowledge’ are actively sought out in a ‘cultural borderland’ (Benor 2012), supported by an ability to integrate common vaccination concerns in the ‘local moral world’ of religious Orthodoxy and pursuit of self-protection.

Simchah (age 45), who migrated from the United States, explained how he was disappointed not to be able to attend the advocacy events, ‘I wanted to go, but no, unfortunately with seven kids—*Baruch Hashem* [thanks to God] with seven kids—it’s just not so easy to get out.’ Simchah and his wife, Atara (age 42), explained how Del Bigtree was used as an authoritative and legitimate information resource to push back against a social pressure to vaccinate in their Orthodox Jewish circles. In his words, ‘it wasn’t that we were pro-vaccination, listened to Del Bigtree, and all of a sudden ‘oh I’m anti-vaccination.’ It was more, ‘I’m anti-vaccination, I need somebody to tell me more why I’m anti-vaccination and to give me some more information so I can counter-argue.’

Nonetheless, Del Bigtree’s vaccine advocacy had a profound impact on the way that Orthodox Jewish parents articulated their perceptions of vaccine damage. Raizel had vaccinated her first child according to schedule and her second child according to a delayed schedule, primarily because of the pressures of having two babies with short periods of spacing. Her perception of vaccinations began to shift when, she said, her eldest child was temporarily unable to walk following the MMR booster at the age of six. Raizel proceeded to draw on Bigtree’s production, *Vaxxed*, to explain what influenced her shift towards non-vaccination:

That’s when the ‘Vaxxed One’ documentary came out, I believe in 2015. It’s from the US, produced by Del Bigtree.^6^ He is the leader along with Robert Kennedy Junior, of the so-called “anti-vax movement,” I would say, “vaccine questioning movement.” He produced this documentary, which went around the states, in a truck asking people to tell their vaccine injury stories. And they were just inundated. So my youngest child is not vaccinated at all. He’s four. I am afraid of the short term and long-term injury risk. There are babies who die right after vaccines, there are kids who are very injured for life, but they’ll [public health] say the ‘science is settled.’ Most *rabbonim* [rabbis] are in favor of vaccines because they don’t research it, they just listen to most doctors, whose portal of knowledge is vertical. The religious reason *not* to vaccinate is *sikun haim* [risk to life]. (Her emphasis)

Thus media informs the vaccine decisions and perceptions of safety risk among migrant American parents, and is used to challenge the position of local rabbinic authorities and health professionals who are otherwise regarded as uninformed. Yet, what also emerges is a convergence of secular and religious truth-claims, as Raizel offers an interpretation of Jewish law to underscore and legitimize her opposition that is rooted in a fear of disability and resolve to protect children from disabilities. Raizel repeats Bigtree’s mantra (‘the science is settled’) to challenge public health claims of vaccine safety, and amplifies the propensity for adverse reactions to cause a universal ‘risk to life’—exemplifying how safety doubt converts into specific Judaic teachings around the body. While she frames vaccinations as contravening religious teachings on avoiding dangers and threats to life, she challenges the authority of rabbis by claiming they are not making independent decisions and have come to take a passive position vis-à-vis medical professionals and not reading into the risk to life that, in her view, vaccines engender.

### Contesting Safety Evidence as Bodily Devotion

Parents such as Raizel and Tehilla opposed vaccination to prevent vaccine injurity or disability, but at the same time under-played the risk of debility from disease. They articulated that their ‘right’ to contract a disease and life-long immunity had been taken away by an over-interventionist state:

I *wanted* my kids to get the diseases when they were little. To me the chickenpox vaccine was the most ludicrous vaccine that ever came out because what’s wrong with chickenpox? It’s a very mild illness. We would have chickenpox parties, me and my friends, and bring our kids together. I got measles. I got mumps. I got chickenpox. Why can’t my children get those diseases? Why shouldn’t they be allowed? It builds their immune systems for life. Vaccines don’t cause life-long immunity. They are man-made and man-given, and disease is God-given. (Tehilla’s emphasis)

Not only are vaccines perceived as ineffective and interventionist, but also a contest over bodily governance—reflecting the discursive production of truth-claims from the advocacy events. Parents who were cautious of vaccination due to safety concerns simultaneously challenged the rhetoric of non-vaccination advocacy. Mordechai (age 56), A Haredi Jewish father from New York, had long held concerns about vaccine safety. He had sought guidance from medical and rabbinic authorities on the dilemma of whether to consent to vaccinating his three daughters or not, reflecting the broader ways that Orthodox and Haredi Jews ‘shoulder moral responsibility’ concerning health decisions (cf. Ivry and Teman 2019). Yet, he too engaged broadly as part of his vaccine decision-making and explained how he was cautious of information sources that amplified the safety of vaccinations without adequately acknowledging the issue of adverse reactions. In his words, ‘I’ve tried to go to the Center for Disease Control website, and they’ll say, ‘vaccines are safe.’ Now it can’t be that simple, so when they sound so condensing and simplistic, that makes me nervous.’ In another breath, however, Mordechai was critical of the information circulated by non-vaccination activists, who, in his words, include ‘those who are religiously anti-vax and tend to portray every disease as being non-lethal and that’s not really true.’ These dissenting voices played out in his own decisions around childhood vaccination:

So we went with vaccination, but I was pretty nerve-wrecked from it. I’m not entirely sure that it didn’t affect them, because one of my kids has developed troublesome allergies. It’s presented as if the only risk from vaccines could be that they might cause autism, but there’s all kinds [of adverse reactions]. This is a sophisticated technology and you’re messing with the body, so I always worried about adverse reactions. But if they, God forbid, got sick from a disease that vaccines help prevent, that also weighs on your mind.

While Mordechai’s concerns reflect the process of vaccine decision-making in non-religious populations (Casiday 2007; Kaufman 2010; Poltorak et al. 2005; Reich 2014), he went on to convert vaccine safety discourse into the letter and language of religious law:

The problem is, I don’t really hear from the medical researchers, who are balanced and objective, faithfully examining the claims by many mothers that ‘my kid was fine, then they had the vaccine.’ I’m largely hearing a lot of nasty rhetoric, and a lot of silencing of people, and whenever I hear silencing, I say ‘wait a second, why isn’t there an open debate? Don’t we want to have all the information out there to make an intelligent decision? We have a religious imperative to take care of our health, that’s the law, but vaccinations are not so simple, we need to ask, “is it safe?” That’s a scientific question. In the Orthodox world, we are willing to question authority, perhaps more than others. We don’t look at doctors as if they’re Gods.

Mordechai invites us to understand how vaccine safety truth-claims or ‘authoritative knowledge’ take shape within a religiously Orthodox ‘local moral world.’ Firstly, Mordechai signifies how the issue of vaccination caused him to negotiate his otherwise self-protective stance, as vaccine safety information is desired and assimilated from the non-Haredi world. He presents having access to diverse sources of vaccine safety information is perceived as necessary to meet the heightened standards of bodily governance that being ‘God-fearing’ entails, which is underscored by Jewish legal codes concerned with preserving health (*pikuach nefesh*). Yet, safety concerns surrounding vaccines clearly point to diverse ways of interpreting the mandate to preserve health or to avoid dangers to health.

Authority is ascribed to knowledge and truth-claims based on a perception that parental claims of vaccine damage and disability are not appropriately investigated by medical researchers. While anthropologists have explored the entanglement of rabbinic and medical expertise in decisions around reproductive biomedicine (Ivry and Teman 2019), the study of vaccination points to a questioning and seizing of authority and ‘authoritative knowledge’ due to the perceived responsibility of parents to protect their children from disability (Kasstan, forthcoming).

Reflecting continuities beyond the case at hand, the public health tendency to dismiss parents’ ‘anecdotal accounts of changes they observed in their children has resulted in many parents feeling that important facts had been overlooked or, even worse, covered up by the medical establishment’ (Casiday 2007:1067). The implications for religious Orthodoxy, however, mark an analytical departure as the construction of evidence on vaccine safety is perceived to have a direct impact on observance of Jewish law and Divine governance of bodies, enabling non-vaccination to be voiced as a right to religious freedom. In the US, parents opposed to vaccines often navigate mandatory laws by crafting claims to religious exemption, though ‘may find themselves challenged by the necessity of claiming a religious belief that they do not actually hold’ (Reich 2018b:232). My data instead capture how vaccine danger is ‘converted’ into a *halachic* or legal issue by Jewish parents opposed to vaccination. It can then be inferred how this conversion process would present implications for accessing religious exemptions to mandatory vaccination in jurisdictions such as the US, where such discursive strategies circulate to and from.

## Discussion

My ethnography demonstrates how the non-vaccination stance of Jewish parents in Jerusalem reflected a concern with safe decision-making, but at the same time, an active engagement with globalized non-vaccination advocacy. Anthropologist Michael Carrithers notes that rhetoric is a ‘penetrating practice,’ where ‘attending to the rhetorical dimension of life requires attending to the rhetorical will, the work on social situations that the persuading agent intends’ (Carrithers 2005:582). Departing from anthropological approaches that examine how technologies travel and gain legitimacy through conceptual transformation (Cromer 2019; Teman, Ivry and Goren 2016; Kahn 2006; Roberts 2006), my approach captures how opposition to biomedical technologies on the grounds of safety circulates and converts through discursive approaches.

Anthropologists have long situated the body as the primary locus of control to reproduce social and political life, constituting a fortified border to protect what is perceived to be pure from what is dangerous (Douglas 2002; Scheper-Hughes and Lock 1987). For this reason regimes of bodily governance are all the more intensified in contexts of religious Orthodoxy. As Nurit Stadler notes, piety forms ‘the only force capable of changing or restraining the secular and heretical nature of the world’ (2009:2). To overcome and circumvent the pursuit of ‘immunity’ and self-protection that characterizes Orthodox and especially Haredi Judaism, Christian non-vaccination activists from the US field a shared vision of Divine governance over bodies and articulate non-vaccination as an act of bodily devotion. Orthodox Jews who oppose vaccination then situate common safety truth-claims in the grammar of Jewish law. Vaccines do not only carry an element of risk for parents, but, as Raizel put it, were *siqun hayim* (a danger to life). Not all Orthodox and Haredi parents, however, agree with the truth-claims presented by non-vaccination activists. Parents such as Moishe feel caught between homogenizing truth-claims concerning communicable diseases on the one hand, and public health claims that vaccines are safe.

In the most extreme form of ‘conversion,’ Christian activists discursively link the ‘technocracy’ (cf. Leach and Fairhead 2007) of vaccination—or state governance over the body—to the Nazi genocide. They present ‘vaccine questioning,’ though in reality non-vaccination, as necessary to avoid a repetition of a devastating collective trauma. Jews should, ‘never again,’ be forcibly subjected to harm by the body politic, a message that becomes assimilated in ‘local moral worlds.’ The circulation and conversion of vaccine rhetoric draws on a historical ‘politics of threatened life’ to engender a conceptual shift from technologies of prevention to technologies of endagerment, thus advancing past debates of how medical risk is situated in cultural scripts of catastrophe (Ivry 2009). Non-vaccination is converted into an act of contesting state authority over the body, and admission of being ‘controlled only by God’—as Del Bigree sought to impart on the American Jewish audience in Jerusalem.

Like any biomedical technology, vaccines are not completely without risk. Parents subsequently navigate safety concerns as a social process when deciding how to most appropriately protect their children’s health (Casiday 2007; Kaufman 2010; Poltorak et al. 2005; Sobo 2015). Non-vaccination activists in Jerusalem framed themselves as supporting the ‘vaccine-injured community,’ yet a critical reading of the rhetoric at play indicates how discursive strategies are premised on a fear of disability and parental responsibility to protect children from disability. Immunity from infection is conceived of as Divine and idealized in ways that overlook the risk of debility, disability and even death from disease. In this regard, secular criticism of vaccine safety is accepted, but voiced as an act of devotion to God and observance of religious law. The parents I met in Jerusalem cross a ‘cultural borderland’ (cf. Benor 2012) as they became Orthodox Jews and migrants, and subsequentltly accept a range of influences into their notions of religious observance.

This study of vaccines reflects how conducts are accepted and appropriated by devotees based on the perception of ‘doing so for God’ (see Taragin-Zeller 2014:76). In advocating for a ‘non-secular medical anthropology,’ anthropologist Elizabeth Roberts places analytical attention on tracing the contingent relationships and ‘material reality of both secular and non-secular sites’ (2016:211). Taking these debates forward, this paper indicates how science-based discourse of risk is articulated in ways that constitute a personal dilemma of religious law. The moral transformation of non-vaccination becomes essential to legitimize counter-positions and to contest pursuits of protection.

My approach lays a foundation for further research to examine the motivations of Evangelical Christians to engage religious minorities in issues of moral regulation. This constitutes a particularly important avenue of research as emerging relations between Evangelical Christians and Haredi Jews appear to have influenced the latter’s responses to coronavirus pandemic control measures in the US (Fader and Berger 2020). In examining the situated ways that global messages surrounding the ethics and safety of vaccines circulate and covert, this paper raises implications for the cultural politics of COVID-19 and how preventative vaccines and possible mandates will be negotiated at local levels.

The empirical contribution of this paper is identifying the rhetorical strategies through which vaccine technologies are presented as an individual and social threat and which work to consolidate vaccination opposition in religious ‘local moral worlds.’ This paper, too, calls for renewed anthropological engagement with how public criticisms of biomedicine circulate and ‘convert’ through encounters with religious Orthodoxy, taking on counter-positions that are versed in the discourse of religious freedom and devotion. Biomedical technologies are a core area of anthropological critique in order to ensure that the highest standards of care are maintained and made available. The ethical commitment that underscores anthropological relationships with participants necessitates an ability to understand concerns surrounding biomedical technology and governance and to call for those concerns to be addressed wherever possible. Incumbent on anthropologists, too, is the task of critiquing how a fear of disability is cultivated and obscured under the otherwise laudable banner of supporting disabled children.

## Footnotes

1 In this paper I examine how non-vaccination activists inspire a conversion of ideas among religiously Orthodox people. For critiques of religious conversion to Judaism in Israel, see Egorova (2015), Kravel-Tovi (2017), and Seeman (1999).

2 *Dati Leumi* Jews are specific to the case of Israel, and typically take a nationalist position based on the integration of Orthodox Jewish and Zionist philosophies.

3 The issue of vaccine safety is a major focus of Bigtree’s advocacy, see for example the ‘Informed Consent Action Network’ (2020).

4 *Ba’alat teshuvah* (feminine), *ba’al teshuvah* (masculine), literally ‘master of repentance.’ The term applies to Jews who transition to Orthodox or ultra-Orthodox Judaism and make a decision to abide by Jewish law.

5 *Eretz Yisrael* is often used in Haredi ‘moral worlds’ as an alternative to the political designation of *Medinat Yisrael* (State of Israel).

6 Bigtree collaborated with Andrew Wakefield, the British physician who controversially claimed that the triple-antigen MMR vaccine is causally associated with developmental disorders in a (since retracted) Lancet article.
